# Information and organization in public health institutes: an ontology-based modeling of the entities in the reception-analysis-report phases

**DOI:** 10.1186/s13326-016-0095-8

**Published:** 2016-09-08

**Authors:** Giandomenico Pozza, Stefano Borgo, Alessandro Oltramari, Laura Contalbrigo, Stefano Marangon

**Affiliations:** 1 Istituto Zooprifilattico Sperimentale delle Venezie, Viale dell’Universitá, Legnaro (PD), 10, 35020 Italy; 2 Laboratory for Applied Ontology (LOA), ISTC CNR, Via alla Cascata 56/C - Povo, Trento, 38100 Italy; 3 Bosch Research and Technology Center, 2555 Smallman Street, Pittsburgh, 15222 Pennsylvania USA

**Keywords:** Ontology, DOLCE, Data transparency, Data integration, Knowledge objects

## Abstract

**Background:**

Ontologies are widely used both in the life sciences and in the management of public and private companies. Typically, the different offices in an organization develop their own models and related ontologies to capture specific tasks and goals. Although there might be an overall coordination, the use of distinct ontologies can jeopardize the integration of data across the organization since data sharing and reusability are sensitive to modeling choices.

**Results:**

The paper provides a study of the entities that are typically found at the reception, analysis and report phases in public institutes in the life science domain. Ontological considerations and techniques are introduced and their implementation exemplified by studying the Istituto Zooprofilattico Sperimentale delle Venezie (IZSVe), a public veterinarian institute with different geographical locations and several laboratories. Different modeling issues are discussed like the identification and characterization of the main entities in these phases; the classification of the (types of) data; the clarification of the contexts and the roles of the involved entities. The study is based on a foundational ontology and shows how it can be extended to a comprehensive and coherent framework comprising the different institute’s roles, processes and data. In particular, it shows how to use notions lying at the borderline between ontology and applications, like that of knowledge object. The paper aims to help the modeler to understand the core viewpoint of the organization and to improve data transparency.

**Conclusions:**

The study shows that the entities at play can be analyzed within a single ontological perspective allowing us to isolate a single ontological framework for the whole organization. This facilitates the development of coherent representations of the entities and related data, and fosters the use of integrated software for data management and reasoning across the company.

## Background

### Ontology

An ontology is the part of the information system that explicitly commits it to a certain conceptualization of the world [1, 2]. When described in terms of lexical semantics, ontologies take the simple form of dictionaries or thesauri; when described in terms of axioms in a logical language, we talk about *formal* ontologies; if logical constraints are encoded in a computational language, formal ontologies turn into *computational* ontologies. Finally, an ontology that concentrates on general and domain-independent categories is called *foundational*.

The applied ontology approach has had a huge impact in all branches of information science. The techniques developed in the last twenty years are now exploited by companies around the world in particular in areas like intelligent interfaces [3], data access [4] and warehouse [5], semantic web standard [6] and medicine [7, 8].

In the life sciences, ontological techniques are applied towards a variety of goals [9] among which the study and organization of areas like genomics [10], anatomy [11, 12], plant anatomical and morphological structures [13], phenotype annotation [14], with important results also in knowledge modeling, organization, integration and exploitation [8, 15, 16]. Notwithstanding these achievements, the application of ontological techniques is still problematic [17].

Our aim in this paper is to show how ontology can be applied to understand the organization and the activity of large institutes woking in the life sciences. We use a public veterinary organization located in north-eastern Italy, namely the Istituto Zooprofilattico Sperimentale delle Venezie [18], to clarify our analysis and exemplify the application of ontological techniques.

### The Istituto Zooprofilattico Sperimentale delle Venezie

The Istituto Zooprofilattico Sperimentale delle Venezie (IZSVe) is an Italian public veterinary institute deputed to conduct prevention, control, research and services in the fields of animal health and food safety. The IZSVe belongs to the Italian National Health Service, is part of a national network that consists of nine other public veterinary institutes, employs more than 600 people (veterinarians, biologists, chemists, technicians and administration staff), and has eleven geographical locations with groups and laboratories devoted to areas like animal welfare, diagnostic services, food risk communication, geographic information systems (GIS), international cooperation, training, veterinary biobank. The IZSVe carries out routine tests in disciplines like diagnostics, virology, parasitology, microbiology, molecular biology and chemistry. Its research activities concentrate on the animal health and food safety fields aiming to develop new diagnostic techniques as well as vaccines and vaccination procedures.

### Limits of standards and software tools

There are many standards centered in aspects relevant to the reception, analysis and report phases of a public institute like IZSVe: the Logical Observation Identifiers Names and Codes (LOINC) [19], the Systematized Nomenclature Of Medicine Clinical Terms (SNOMED CT) [20], the ISO/TC 212 Clinical laboratory testing and in vitro diagnostic test systems and others [21]. Also, there are several languages and conceptual modeling techniques that the knowledge engineer can use to develop information systems, e.g., the Business Process Modeling Notation (BPMN) [22] or the Unified Modeling Language (UML) [23] and its related standards like the Model Driven Architecture (MDA) [24]. However, all these standards and modeling systems focus on some elements, e.g. LOINC, or aspects, e.g. BPMN, of the complex scenarios in public institutes like the IZSVe. Even the UML language, which is perhaps the most broadly applicable, assumes that the modeler adopts a viewpoint, i.e., has an understanding of the scenario. How to reliably understand the domain and, more specifically, the scenario of interest is a goal explicitly addressed by ontological analysis.

Regarding the analyses carried out in laboratories, typically a Laboratory Information Management System (LIMS) is exploited to manage and coordinate the information on the tests and the related set of materials and procedures. Nonetheless, the management of a large set of laboratories and tests is quite complicated and in many cases the LIMS is applied to the subset of data that are homogeneous across laboratories and activities. This is the situation at the IZSVe, which offers about 950 types of tests and runs almost 1.7 million tests per year. It has been recognized that having a partial management via the LIMS reduces the possibilities to coordinate, monitor and analyze the institute’s activities and the performance. On the other hand, the adoption of different LIMS allows to take into account the specificity of each laboratory. However, fine tuning each LIMS to a specific case jeopardize the possibility of an integrated data and process management system.

These problems arise from a substantial lack of a unifying understanding of the institution’s scenarios and of the role that the different elements (objects, tools, data, personnel) play in its activities. We study this issue looking at the IZSVe’s use case. The goal is to develop a global view for a centralized management system and verify the contribution of ontological techniques in understanding complex situations.

The ontological approach is also promising in dealing with the information system’s evolution. The IZSVe adopted a state-of-the-art LIMS ten years ago and, since then, the system have gone through several updates, revealing a certain lack of flexibility. It is recognized today that the LIMS is unable to evolve with and adapt to the company’s needs. It is unclear whether the problem lies in the technical and implementation aspects of this LIMS or in the initial analysis of the institute. Most likely, it is a combination of both. In any case, to align the LIMS and the institute’s activities, a deep understanding of the institute seems necessary, including its reasons to exist and its strategies. This information is about what the institute does and why, and should not be confused with how the institute does it today or at any other period of time. These are inherently ontological distinctions.

### Ontological analysis

Some of the most relevant issues posited by the scenario we study are: (a) to identify and characterize the elements in the institution’s activities; (b) to classify the types of data that are needed and to identify where they are used; and (c) to make explicit the context and the role of the involved entities.

To study the IZSVe scenario we adopt an approach based on a foundational and formal ontology. The use of a foundational ontology ensures that the principles are not constrained by the specific domain we work with which, in turn, leads to an understanding of the institute independent from contingent settings (like the organization of the institute at some point in time or the set of tests it makes available). This feature makes the resulting model more flexible. For example, the introduction or modification of other laboratory methodologies and techniques, which requires important changes in traditional rule-based systems, is managed in an ontological model via extensions, e.g. by adding (or dropping) classes and their descriptions. Furthermore, the use of a logic language with formal semantics allows to check the consistency of the system well beyond approaches like BPMN and UML.

In the rest of the paper we use ontological analysis and a foundational ontology to study the reception, analysis, and report phases in public institutes in the life science domain. The goal is to show how to understand the scenario, how to isolate and distinguish the relevant entities, to indicate their relationships and which roles they play. The entities we discuss, like specimens, reports, sample seals, laboratory tools, data sets, administrative stuff and so on, will be classified according to their ontological types.

One important result of this work is the development of a single framework where it is possible integrating all the discussed entities, data and roles. Working with a single model helps also to evaluate the coverage of the domain, to check the coherence across elements and phases, and to facilitate maintenance. Finally, as pointed out earlier, by using a foundational ontology we expect that the model we obtain will remain valid across time, provided the essential constraints remain unchanged (e.g., in the scenario, the law defining the IZSVe’s institutional goals).

## Methods

In this part we first introduce the ontological analysis approach and give some indication on how its application can be evaluated. Then, we use aspects of the IZSVe scenario to introduce the rationale, the basic structure and some categories of the DOLCE foundational ontology. Later, in the next section, we will use ontological analysis to discuss typical modeling issues taken from our scenario, and will use the results of this analysis to expand the DOLCE ontology to an ontology for public institutes in the life sciences.

### Ontological analysis and its assessment

Generally speaking, it is important to distinguish how things occur in the activities and what are the expectations we have about them. Ontological analysis helps to make subtle, yet crucial, distinctions. For example, ontological considerations lead to separate the seal of a specimen container as a signal of integrity (a role) from the seal as an artifact (an object). Once we have identified the entities in a scenario, we need to organize them in a hierarchy and to establish their relationships (e.g. it is the object-seal that plays the role-signal of integrity, and stops when broken), and to ensure that the hierarchy leaves space for future extensions. After all, a finer analysis may lead to introduce new entities, and one may want to expand this very hierarchy on aspects not yet considered like new procedures, safety regulations, responsibilities or workload management.

Usually, a model presents a perspective. It can present the scenario from the perspective of the service user, from that of the overall organization, or from that of a sample to be analyzed. Since all the key elements that characterize these different tasks and views could be organized within an ontological framework, we aim to show that it is indeed possible to construct such a general framework. This framework provides a conceptual system that comprises the different perspectives and, thus, is suitable for tasks as different as data management, quality assessment and responsibility tracking. Our work is based on techniques that have been developed from the 90s to guide the development of robust ontologies, see e.g. [25–31].

Being a conceptual tool, ontological analysis is not suitable for quantitative evaluation. However, there are different qualitative parameters to assess its results, e.g. [32]. We will apply them to evaluate the results we obtain in studying our guiding scenario (see Sections Devising the ontology and Framework Evaluation).

### The IZSVe scenario

All the IZSVe laboratories activities related to sample receiving, delivering and transferring are coordinated by a centralized delivery service, the Reception and Public Relation Laboratory (LARU), located at the headquarters. All the information on the samples and the analytical processes (like tracking the samples distribution and managing the analysis procedures) are registered in the dedicated LIMS, called IZILAB. IZILAB manages also data unnecessary for the tests, e.g., information about involved parties (sample deliverer, sample owner, sample collector, etc.), reason of investigation, submission form number, breeding or food processing plant where the sample was collected. Data are collected from different sources from human operators to automatic access to, e.g, the farm registry database.

The rest of this section presents a typical IZSVe scenario. To keep the presentation simple, the description focuses on some interesting parts of the overall system. Later we will refer to this scenario as our “guiding example.”

A qualified technician, personally or via a delivery service, delivers a sample – e.g., a pathological specimen from alive or dead animals or food for human consumption – to a IZSVe reception point requesting to test the specimen on some characteristics, e.g. microbiological safety. The reception unit personnel collects the sample, the submission form with the required analysis and performs some preliminary check like the presence and integrity of sample seals; the sample’s storage temperature (when needed); the match between the declared number of samples (or units) and the delivered items; the presence of the requested analysis in the list of services provided by IZSVe.

The reception unit may perform more specific checks to verify, for instance, that: the needed information is reported in the submission form; the form lists any special management constraint (e.g. the analysis might be requested at a fixed date and time for the participation of external observers); the sample has been correctly collected and preserved for the requested analysis type (e.g. storage temperature; timing of the analysis).

After the checks, the sample is registered in the IZILAB and unique identification labels are physically attached to the sample container and to the analysis submission form. A receipt with the identification number is released to the deliverer. From this point on, the IZSVe is fully responsible of the sample management and all the data needed for the IZSVe internal procedures are made accessible to the operators via the IZILAB.

**Fig. 1:**
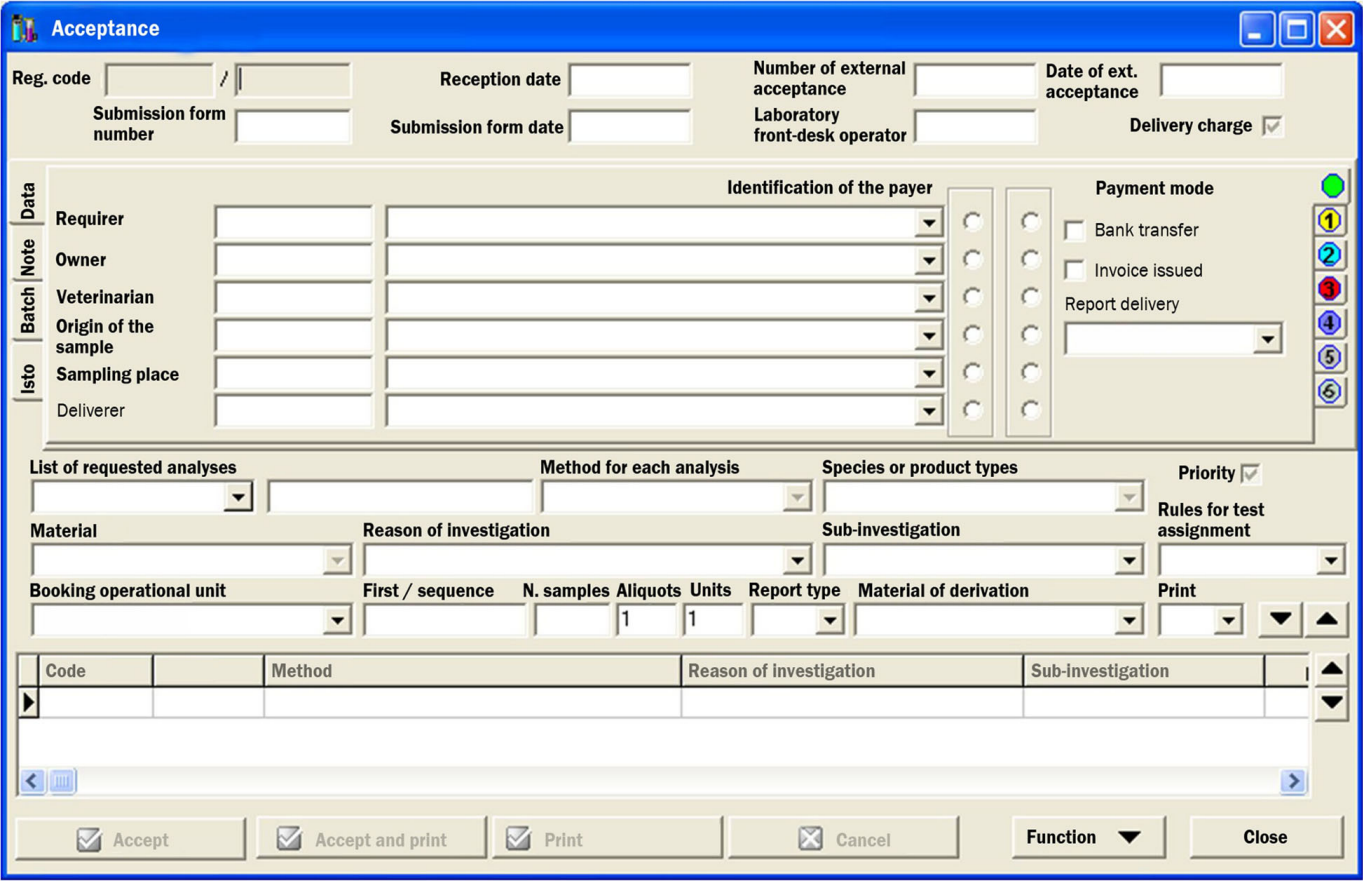
GUI at the lab’s reception. Graphical user interface (GUI) translated, original in Italian

Next, the sample is stored in a ward (storage room, cooler, freezer) to be distributed to the laboratories. The registration data, called “batch”, is added to the IZILAB’s “batch-list” of the ward (a kind of loading/unloading register). The sample is then collected by laboratory personnel or delivered via the IZSVe service. At the laboratory, the administrative and technical staff make a final assessment on the suitability of the sample for the requested analysis: the documents and the compliance of the specimen to the test requirements are verified. Then, the seals are broken and direct inspection of the sample content can be done. The laboratory personnel complete the data via a dedicated interface in IZILAB: the batch code ensures that data are added to the right record as well as the consistency of the sample tracking information. Some fields are shown in the IZILAB lab’s reception GUI - Graphical User Interface -(Fig. 1). The “number of external acceptance” and the “date of external acceptance” are needed to coordinate further tests, if any, run by other IZSVe Laboratories. The flag “delivery charge” activates the fields for delivery charges. The flag “identification of the payer” indicates the party charged for the procedure costs. The “rules for test assignment” provides information on the acquisition of digital data while the “first/sequence” is needed when there are several samples and/or several analyses are done on the same specimen.

Once the data are uploaded in IZILAB, a working paper (lab sheet) is prepared where the lab technicians report the steps of the analysis process. At the end of the analysis, the administrative assistant uploads into IZILAB the rough data and the Laboratory Head checks whether further tests are needed, e.g. for verification. If s/he decides for further tests in a different laboratory, the process (transferring and analysis) is repeated as before but the “batch-list” (loading/unloading) is now linked to a “waiting acceptance list” devoted to samples moving from lab to lab. Once the analyses are completed and all data are collected in IZILAB, the report is produced by the administration and the Laboratory Head digitally signs it. The file is then made accessible (in full or limited form) through the web to the parties that have the right to access it.

### Devising the ontology

Research in applied ontology has devised a series of logic-based relations and categories that furnish the starting point for entity analysis and classification. The idea is to explicitly list all the types of entities that are taken to exist (or at least to be of relevance) in the application domain and to classify each specific item as belonging to one single category. Further constraints help to enforce the right use of the hierarchy.

**Fig. 2:**
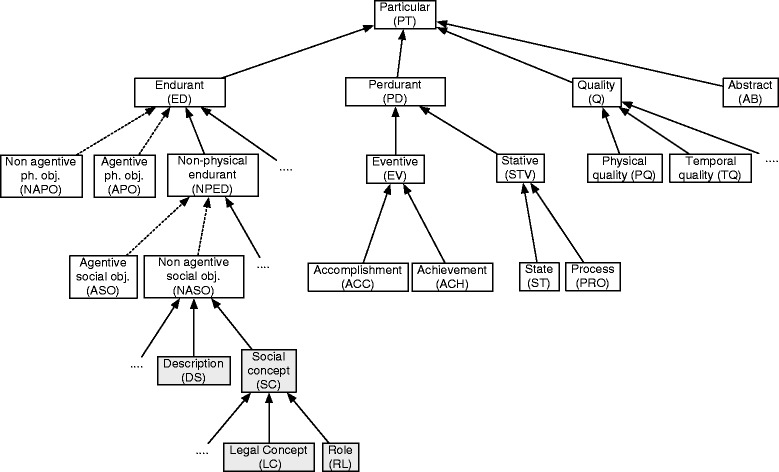
Category hierarchy of the DOLCE ontology. DOLCE fragment, from [33], with an extension of the social object category (gray boxes). *Arrows* represent ISA relationships and dotted arrows chains of ISA

In this paper we adopt the foundational ontology DOLCE [33] with some extensions relative to the categories of roles and descriptions as presented in [34]. DOLCE is a foundational and formal ontology developed from cognitive and linguistic considerations and with particular emphasis on social reality (Fig. 2). Our choice of DOLCE relies on a few observations: DOLCE’s underlying principles and construction techniques have been well described [33] and there is evidence that this ontology is preferred even by non trained users [15], the ontology is available in different formalisms [35], it is stable and several extensions are available, e.g. social roles [36], artifacts and products [37] and mental states [38]. Furthermore, the ontology has been verified in terms of ontological and logical soundness [39, 40].

Here we describe the parts of the ontology structure, and some of the basic relations, that are needed to understand the material in this paper. The presentation is minimal, the user can find a complete introduction in [33, 34]. DOLCE is based on the basic distinction between objects, events and qualities. Other important categories are those of descriptions and roles. Objects (DOLCE category: *Endurant*) are entities that are mainly identified by their relationship with space while they persist in time. People, samples, laboratory equipments as well as the cavity of a specimen container are classified as objects in DOLCE. Events (DOLCE category: *Perdurant*) form a different category; these are things that necessarily happen in time like the (process of) delivering a sample or running a laboratory test. The category *Quality* gathers individual properties, i.e. properties associated to a specific object or a specific event, and that serve to qualify them: each sample has its own specific weight and temperature, each instance of a laboratory test has its own duration etc. Qualities can be simple like weight, temperature and duration; or complex like price, frequency and speed. The category *Description* collects information entities, like procedure specifications, and the category *Social Concepts* entities like the Italian and the European legal notions of organization. We anticipate that members of the last two categories are generally seen as temporal objects (they live and change in time) but for practical reasons they are here treated as atemporal entities. Thus, here we ignore that the society, the laws and the concepts evolve in time. This choice will become clear in Section Roles and players in the IZSVe scenario. Descriptions and social concepts are distinguished in the ontology from their physical supports and realizations: the EU legislation on the control of the notifiable disease of livestock (e.g. Foot and Mouth Disease) is ontologically a concept, thus a different kind of entity than the document where it is written (which is a physical object), and the application of the regulation is still another kind of entity, namely an event. This event is the instantiation of a procedure, which is a description. A further class, *Role*, collects properties that identify a temporal status of an entity usually dependent on some social contexts [36, 41, 42]. For example, an individual agent may play the role of IZSVe’s Laboratory Technician in some period of time. Similarly, a physical object can play the role of a Sample or of a Seal within a IZSVe activity (which provides the context). A fragment of the DOLCE categories, with the mentioned extensions, is presented in Fig. 2.

Our goal in the rest of this paper is to expand the DOLCE framework with new categories tuned to the modeling issues elicited from scenarios like that of IZSVe. This activity is known as “domain adaptation” [43] and aims to cover the notions characterizing the application domain.

The relations among the categories must also be fixed. Structural relationships, like subsumption (aka ISA), instantiation and parthood [29] are already part of the DOLCE language. Subsumption is the subclass relation: for instance, the category of *Agentive social object* (ASO) is subsumed by the category of *Non-physical object* (NPED), Fig. 2. This means that any ASO entity, e.g. a company, is also an NPED entity. Similarly, the IZSVe category *Sub-analysis* is subsumed by the IZSVe category *Analysis*: any IZSVe sub-analysis is also a IZSVe analysis. The instantiation relation applies to a class and an individual, it states that the individual is an instance of the class. As an example, the object identified by code PD/A123 is an instance of the category *Sample*. Note that, being this latter category a subclass of *Endurant*, the object identified by code PD/A123 is also an instance of the category *Endurant*. This result is a consequence of the formal interaction between the instantiation and the subsumption relations. Finally, the parthood relation is used to state that an entity is part of another entity, e.g. report *X* of laboratory *A* can be part of report *Y* of laboratory *B* (say, when the first reports about a related analysis on the same specimen). The example needs clarification: report *X*, in the sense of a description (a collection of data, thus an information entity), is part of report *Y*, also understood as a description. On the other hand, report *X* in the sense of a piece of paper (or an electronic file) is part of report *Y* provided now we understand even *Y* in the sense of a piece of paper (or an electronic file). The ontology tells us that no other combination of these senses holds. The use of a formal language, as in DOLCE, helps to keep these two parthood statements apart and to correctly relate them the right meaning of the terms. Similarly, parthood can be used to state that an instantiation (a specific event) of procedure A (a type of event) is obtained by the (mereological) sum of instances of procedures *B*_*1*_, *B*_*2*_ etc. We refer the reader to [33] and [29] for further details on these relations.

## Results and discussion

In this part we exemplify the use of ontological analysis via the IZSVe scenario that leads to the classification in Table 1, introduce other relevant notions and then evaluate the results. We pay particular attention on the selection and description of the static elements and in particular on roles and dependencies across entities. The discussion of events and their interrelationships aims to highlight the variety of distinctions that can be made. A comprehensive analysis of events from the ontological viewpoint is out of the scope of this paper.

**Table 1:** IZSVe elements in our guiding example

Cooler	Analysis report	Batch-list
Laboratory equipment	Freezer	IZILAB
IZSVe	Laboratory report	Laboratory room
Receipt	Sample	Sample reception point
Sample label	Seal	Submission form
Storage room		Working sheet
Waiting the acceptance list issue	Awarding of the batch number	Booking of additional tests
Checking the rough data	Checking the sample	Colleting the sample
Filling the submission form	Delivering samples to the laboratory	Delivering the sample to the LARU
Issuing the receipt	Editing the batch list	Editing the report
Signing the test report	Breaking the seals	Recording the data
Registrating the sample		Requesting the analysis
Storage temperature	Accuracy of a test	Cost of a test
Priority of a test	Duration of a test	Integrity of seal
Sample temperature	Repeatability of a test	Reproducibility of a test
Number of aliquots of a sample		Number of steps in a procedure
Working sheet content	Analytical method description	LARU procedure description
Laboratory procedure description	Laboratory result for a sample	European (national etc.) procedures
*Sample Owner*	*Administrative Assistant*	*Deliverer*
*Requirer*	*Frontdesk Operator*	*Report Receiver*
*Head of Laboratory*	*Laboratory Technician*	*Qualified Technician*

### Categories and participants: a look at the IZSVe scenario

- *Endurant.* The objects are grouped via new subcategories of the DOLCE category *Endurant*. We introduce categories of documents like the analysis result form and the analysis result (an analysis result form is a document suitable to list the data obtained from an analysis procedure, the analysis result is the document filled out with the data), the analysis request form and the analysis request, the registration form and the test report. Other objects are person, organization, agent, laboratory tool, laboratory material, container, building etc.
- *Perdurant.* The new types of event are subcategories of the DOLCE category *Perdurant*. We focus in particular on perdurants identifying activities like: running a laboratory test; disposing, storing, transferring and delivering a sample; preparing, signing and delivering a test report; requesting an analysis; breaking a seal etc. Among these, activities like running a laboratory test and disposing a sample, have a clear ending point (these activities are called accomplishment in DOLCE). Others, like storing a sample, do not and are called states.
- *Quality.* Qualities in the scenario are divided into a variety of subcategories. Beside the usual qualities like weight, shape, duration, speed etc. we model also accuracy, reproducibility and repeatability of the tests, price and priority as individual or relational qualities.
- *Description.* The category *Description* is a subcategory of *Non-agentive social object* and collects social entities that are neither agentive nor material. These are information objects that serve as classifiers for other entities. Typical examples are descriptions of a tool or a specimen (descriptions of endurants, for instance the data associated to a specimen), descriptions of specific processes or actions (descriptions of perdurants, for instance a description of how specimen *X* was delivered to laboratory *Y*) and descriptions of methods (descriptions of rules and other constraints, for instance the description of the procedure for specimen delivery).
- *Social Concept.* This is a subcategory of *Non-agentive social object* closely related to the previous (see Fig. 2). It is populated with information objects that acquire social relevance. Here we find the content of official regulations and laws, or the data produced by an official test (these data, legally binding or not, give always a description in the sense of the previous category). Other descriptions with a binding social value, like the official description of a test procedure, are in this category. Two subcategories of *Social Concept* are particularly relevant to our work: the *Role* and the *Legal Concept* categories.
- *Role.* We focus on two types of roles: agent roles, played by agentive entities only (typically humans or organizations), and functional roles played generically by non-agentive physical objects. Among the agent roles there are laboratory technician and laboratory head, sample deliverer and analysis payer. Among the functional roles, we have specimen, reagent, seal and reference material (note the distinction between the material, typically an amount of matter or an artifact, and its role in a procedure).
- *Legal Concept.* This category is here introduced following studies in the legal domain [44]. It collects European, national, regional and internal laws and regulations intended as sets of normative specifications, that is, not mere descriptions nor generic social concepts.
- *Abstract.* The category of abstract entities does not seem relevant in this context. We mention it just to remind the reader that it serves to classify entities like numbers and quality spaces [33].

These are the most relevant categories in our specific scenario. More specialized categories are discussed later in this section. Although the overall scenario is really rich, these categories exemplify all the issues we found and suffice to clarify the key modeling choices in this scenario.

### The notion of knowledge object

Due to historical, legacy and business factors, organizations may adopt special perspectives which are not ontologically justified [45]. For instance, the IZSVe considers a type of object that is actually a mix of three DOLCE ontological types: a material entity (e.g. a sample), an information entity (the sample information in IZILAB) and an agent role (e.g. the personnel in charge of the sample). This mix defines an ‘official sample’ which is a crucial element in the institute’s legal activities. This situation is fairly common across companies although the characteristics of these entities may change considerably. Since these entities are important for the organization and are not ontologically consistent (an ontological entity cannot be member of disjoint categories), to model them we use a specific methodology [46, 47] allowing to introduce a new type of objects called *knowledge objects*. The extension is not necessary for the organization’s information (the ontology suffices for this goal) but helps to include in the model these special perspectives of the organization.

Let us see how knowledge objects can be modeled by studying the IZSVe case. Given the above description, an IZSVe knowledge object *KO* comprises a material entity *M*, an information entity *D* and an agent role *A*. Then, the object *KO* is the triple (*M,D,A*) such that at time *t*, the element *D* of *KO* collects the information that IZSVe has at that time *t* on *M* and the element *A* is an IZSVe role responsible of the status of and the changes in *M* and *D* at that time. Typically, the *M* is a physical object or a quantity of matter officially delivered to IZSVe for analysis. The entity *D* is created when at the time of the *M* reception an IZSVe operator creates the data record about *M* in the IZILAB. Thus, *D* is the information object relative to *M* stored in the IZILAB database and regularly updated during the IZSVe’s procedures on *M*. *A* is the person responsible of the service or laboratory that is managing *M*.

**Fig. 3:**
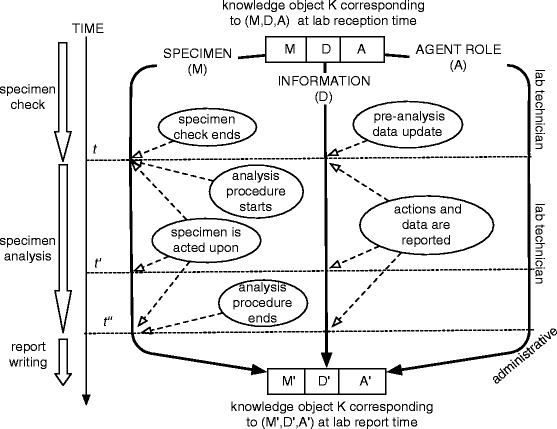
Tracing the changes in IZSVe’s knowledge objects. Possible changes that a knowledge object undergoes from (*M,D,A*) to (*M* ^′^,*D* ^′^,*A* ^′^) within the IZSVe scenario

Since knowledge objects are not part of the ontology, they should be seen as auxiliary elements of the information system. They evolve in agreement with their ontological components (*M*, *D* and *A*). A sketch of the changes that a knowledge object *KO* undergoes from its status at the reception time *t*, given by (*M,D,A*), to its status at the time *t*^′^ in which the laboratory report is written, indicated by (*M*^′^,*D*^′^,*A*^′^), is shown in Fig. 3. The figure highlights three temporal points *t,t*^′^ and *t*^″^, and three phases (time is oriented top-down): specimen check, specimen analysis and report writing. The solid arrow on the left is marked by events relative to changes in the material component *M*, the solid arrow in the center is marked by events relative to the information component *D*, and the one on the right to the agentive component *A*. *KO*, as a whole, has also its own specific properties. We say that a *KO*=(*M,D,A*) is *complete* whenever *D* contains *all* information relative to *M* that are of value for the IZSVe’s activities and institutional goals. Furthermore, the procedure to manage the *KO* is said to *preserve correctness* if, given that the data in *D* relative to some property of *M* at a time *t* is true, then at any later time *t*^′^*D* contains only true data relatively to that property of *M*.

Recall that, due to the introduction of knowledge objects, the model we are building is not purely ontological. The reason is that these knowledge objects, being a combination of elements from disjoint categories, rely on a combination of properties that is incompatible with the assumptions of the ontology. In the ontology *M*, *D* and *A* are separated elements interconnected via dependency relations (*D* contains information about *M*, *A* is responsible for *D* and *M* etc.). The corresponding knowledge object *KO* is ontologically understood as a set of cross-categorical constraints. If there is a need to include knowledge objects within the ontology itself, one can apply the reification technique presented in, e.g, [30].

### Processes in the IZSVe scenario

The categories previously discussed allow to talk about the organization’s activities, the flow of information in the different phases and the participating roles. Ontology can be extended to model finer information.

**Fig. 4:**
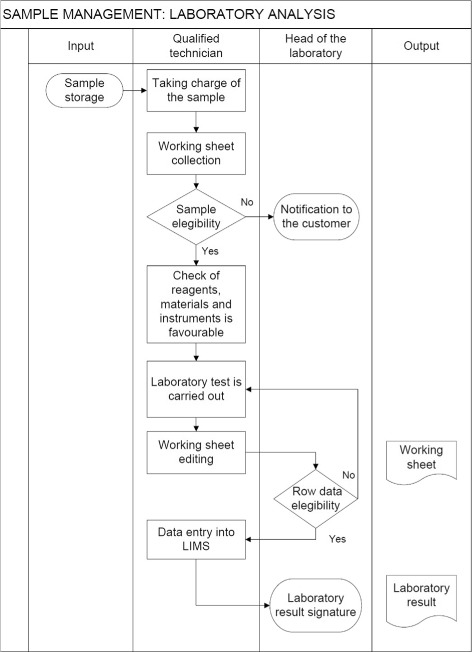
Partial flowchart of the IZSVe’s scenario - Laboratory. Laboratory technician and head views only

For instance, take the flowchart (ontologically this is a description) of the IZSVe laboratory analysis phase (Fig. 4), indicating the roles of the lab technician and the lab head. The figure takes the viewpoint of the sample; the graph lists the set of actions following the temporal order and some causality constraint.

This graphical representation is weak from the ontological perspective since it hides distinctions that can be important for, say, management tasks. Ontology analysis tells us that a generic event is a bundle of more specific events: the stable properties (these identify a state in DOLCE); the dynamic regularities (a process in the DOLCE’s terminology); the evolving conditions that lead to complete the activity (an accomplishment in DOLCE); the key transition moments (an achievement in DOLCE). Thus, ontologically an activity like the data entry into LIMS (see Fig. 4) comprises several types of events that we might want to distinguish, for instance: the continuous relationship between the hardware, the software, the work location, the data and the operator (state); the evolving input/output interactions between the operator, the database, the program and the hardware (process); the steps that lead to the update of the information stored in the database (accomplishment); and the event of the instant in which the new data is physically recorded in the database (achievement).

### Roles and players in the IZSVe scenario

The role category is characterized by dynamicity (one can play a role just temporarily), anti-rigidity (playing or not a role does not change the entity’s ontological status) and relational dependency (a role depends on external definitions, its context) [33]. Other views exist: [48] separates the role hierarchy from the category hierarchy so that a (specific) student depends on a (specific) person but is itself not a person; [49] sees roles as realizable dependent entities that fully exist only while they are played; and [42] takes the relationship role-context as primary.

**Fig. 5:**
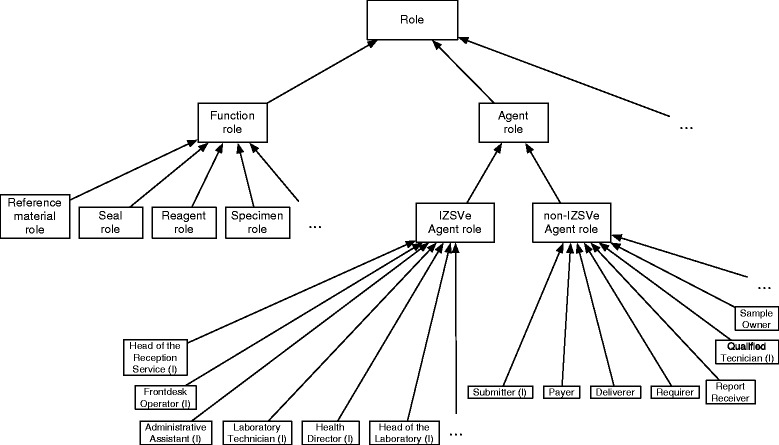
Some roles in the IZSVe scenario. Major roles of the scenario in Section Methods, “I” indicates individual roles

Notwithstanding the differences, to model the social reality we need roles. Agent and actors are different things: an agent is an entity that can act, an actor is an agent whose acts acquire a social value. There are also roles played by non-agentive entities: a quantity of biological material can play the role of a specimen (Fig. 5). On the other hand, not all the distinctions are equally relevant. For instance, do we need to distinguish between a specimen container (the artifact) and that very object when used as a container in a laboratory (the role)? Ontologically, these things are kept apart and, yet, *in the modeling context* the distinction is negligible. Thus, we suggest to have the two notions in the ontology but organize the information system in such a way that it uses only one (typically, the role category). Unfortunately, we lack more precise guidelines on this issue (see also Section Functional roles).

#### Agent roles

We separate roles that act for the company, in our scenario these are called IZSVe *internal roles*, from the others, here called IZSVe *external roles* (non-IZSVe roles, for short), see Fig. 5.

**Fig. 6:**
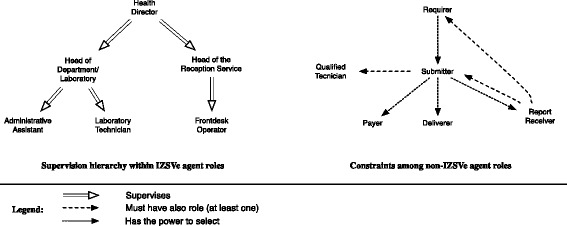
Constraints on agent roles in the IZSVe scenario. The supervision relation among the internal roles is standard

The internal roles are components of the company and are played by its personnel: all these roles must be played by individual agents and are thus marked by “I” in Fig. 5. The scenario involves six internal roles, namely, *Health Director*, *Head of Laboratory*, *Head of the Reception Service*, *Laboratory Technician*, *Frontdesk Operator* and *Administrative Assistant*. The relationship of supervision holding among roles is quite standard in today’s social organizations and we do not discuss it further (see “Supervision hierarchy” in Fig. 6).

The non-IZSVe roles are mentioned in the company’s procedures but not structured within its organization, namely: *Sample Owner*, *Submitter*, *Payer*, *Report Receiver*, *Deliverer* and *Requirer* (Fig. 5). We find that another role, here called *Qualified Technician*, needs to be added to the list although it is never introduced in the IZSVe scenario. A *Qualified Technician* is a role that can be played only by individuals with a specific degree, e.g. a veterinary title, and registered in a dedicated public repository. This role is needed to justify the social power of the *Submitter* role. Also, the *Submitter* and *Qualified Technician* are the only non-IZSVe roles that must be performed by an individual agent. In all remaining cases the player can be an individual agent or an organization. Four of these roles, namely *Submitter*, *Report Receiver*, *Payer* and *Deliverer*, are the interfaces between IZSVe and the external social system: the *Submitter* makes the official analysis request and thus triggers the IZSVe activity; the *Report Receiver* is the receiver of the service report, the *Payer* is needed for the economic sustainability of the service, and the *Deliverer* (which physically delivers the sample to the IZSVe reception site) takes care of the physical interactions. It is interesting to note that the *Sample Owner* role, the only non-IZSVe role remaining, is not a figure that has a direct relationship with the IZSVe. This is also seen by the fact that its involvement is not motivated in the scenario. These specific cases are due to the institutional goals of the IZSVe: to collect data on the regional territory and to anticipate possible problematic situations. When there is a suspect of potential food contamination or presence of a disease, knowledge of the *Sample Owner* allows fast reactions by the authorities.

Differently from the internal roles, data on non-IZSVe roles might be missing from IZILAB. This lack of information is a factual issue, not a modeling problem. The IZILAB system requires explicit knowledge only of some roles: *Submitter*, *Report Receiver* and *Payer*. For example, the *Deliverer* role is marked “optional” in the IZILAB GUI. Since the analysis request form is delivered *together* with the sample, the information about the *Deliverer* was considered marginal. This, however, shows that the IZILAB designer did not have an integrated view of the goals/duties of the different roles. As of today, nothing prevents the (player of the role) *Sample Owner* to play the *Deliverer* role as well. But the two roles may have conflicting interests: the *Submitter* trusts the *Deliverer* to correctly manage the sample delivery so that it can be correctly tested. Yet, the *Sample Owner* could be interested in altering the sample (e.g. not following storing requirements) to prevent the possibility to test it correctly. Our analysis suggested to better model the roles’ interrelations so to prevent these cases, possibly enriching existing guidelines.

Our analysis shows that all IZSVe external roles are mutually independent with two exceptions. (1) The *Submitter* role must also play the *Qualified Technician* role, as we have already discussed. (2) The *Report Receiver* role must be played by the player of either the *Requirer* or the *Submitter*.

#### Functional roles

This is the other subcategory of roles we deal with in Fig. 5. Here it is important to understand the artifactual and the contextual status of objects. A laboratory tool is an artifact manufactured to realize some functionality, a specimen is a quantity of (natural or artificial) material selected as “representative” of some substance or object. A general approach for artifactual entities and their roles is presented in [42] where one can model a lab container when used as such or when used as, say, a pencil case. Indeed, the lab container artifact may play a role for which it was not produced. Unfortunately, this approach is based on the notion of context which is hard to model [50].

The study of functional roles in the IZSVe scenario leads to many subtle distinctions, which may be sensitive to the granular level of the description [51]. We model the artifactual status of the entities via the notion of ontological artifact [37] and technical artifact [52]. (Alternative ontological views, e.g. [53], could be easily adopted.) Entities like equipment and laboratory material, are always playing the intended role in this model since the IZSVe organization and its scenario are quite rigid on this. Note however that where different levels of granularity are needed, one should not simplify the model in this way. For instance, we classify specimens, seals and reagents as roles (Fig. 5) but treat laboratory tools and containers as endurants, see Section Categories and participants: a look at the IZSVe scenario.

Of course, a specimen is an artifact in the sense of [37] since it has been intentionally selected and it has the (intentionally attributed) capacity to provide information about the whole material from which it is extracted. However, in the IZSVe procedures specimens may have different status. This happens in particular when the dependencies between the components of the corresponding knowledge object (section The notion of knowledge object) are broken, e.g. when a wrong or incorrect procedure is applied or the responsibility chain is broken. In these cases, the specimen looses its “official” or legal status. To make room for this change, we include both the specimen as an artifact and the specimen as a role. Similarly, when one entity enters in the scenario as a seal, it does so to guarantee the integrity of the container. Once the seal is broken, the seal looses its role and thus changes its status, it is still a seal from the artifactual perspective but it is not “sealing” any more. Similar arguments apply to the modeling of reference material and of reagent (before and after their use). In contrast, entities like a registration form, a test report and a laboratory tool (in the sense of non-consumable tool like a microscope) maintain their status throughout the IZSVe activities independently on what happens. Note that this leaves out from the model events that destroy the object’s functionalities (beyond malfunctioning).

### Framework Evaluation

The evaluation of an ontology developed for application scenarios is still largely debated in the literature [32, 54–56]. Among the different criteria listed in [32], our work aims to: (a) reach an agreement about meanings of terms in a vocabulary, (b) provide a uniform view to facilitate data integration across distributed sources, and (c) develop a formal model that allows automatic verification of its own consistency and accuracy. According to the analysis in [32], the use of foundational ontology increases coherence and interoperability; the provision of unambiguous and formal documentation increases coherence and clarity; the provision of machine-readable documentation allows for automated data processing, automated knowledge- and data-integration, semantic integration; consistency verification helps to detect modeling errors and increase data coherence. Since we focus on modeling methodologies and ontological analysis in existing complex scenario, we will concentrate on the conceptual analysis, reusability and consistency criteria. The model is in the design phase and has not been implemented. User feedback is limited and restricted to people that have been involved in the scenario analysis or interviewed to describe the IZSVe phases. Unfortunately, these data are limited and not suitable for evaluation methods like statistical analysis.

Following today’s practice [32], we listed five parameters to evaluate the result of the application of ontological analysis, namely: *generality*, *openess*, *flexibility*, *coherence* and *consistency*.

1. *Generality.* As discussed in Section Devising the ontology, we started from an existing ontology, DOLCE, which has been deployed in domains as different as engineering, finance, fishery and medical image analysis. This previous experience indicates that DOLCE is comprehensive, conceptually sound and not focused on any particular perspective of the scenario. Also, other independent evaluations, e.g. [39], established that DOLCE is comprehensive and suited for mesoscopic entities, that is, commonsense entities cast by human reasoning and language, and that are at the center of our social environment.
  In extending the ontology to cover the IZSVe domain we have followed the DOLCE principles and construction methodology. This approach ensures two things: no new restriction is added to the system, and the different areas (data management, laboratory conduct, responsibility hierarchy, resource administration etc.) are or can be included in the ontology. Since the new categories have an auxiliary role and do not form partitions, our extension inherits the generality of DOLCE and preserves it.
2. *Openness.* The proposed extension of DOLCE models domain notions like specimen, laboratory tool and method description. This is obtained by introducing specialized categories and by populating them without introducing new partitions or cross categorial constraints on DOLCE itself. It follows that further categories can be added at each taxonomical level of our extension. For instance, a new category collecting the roles related to some other process (e.g., contract management) can be added to the IZSVe roles without having to revise the ontological system already developed. This design choice ensures that the system is open to revisions and further extensions.
3. *Flexibility.* Flexibility is obtained by balancing ontological assumptions and formal constraints. Our extension adds a new set of domain-dependent properties to characterize the new categories. In some cases, e.g. for knowledge objects (Section The notion of knowledge object), we departed from the DOLCE perspective and applied a methodology to model entities not classified by the ontology. Our approach ensures that this new kind of information is managed in the ontology as a set of requirements or dependencies. This choice allowed us to mediate between the strict ontological sieve and the more permissive attitude one has in applications. Finally, the constraints introduced via knowledge objects are local in the sense that they apply to only those entities that are connected via the notion of knowledge object. As such, these constraints do not limit the ontology itself.
4. *Coherence.* A foundational ontology like DOLCE provides a unifying view within which one can identify and classify every entity in the domain of study. This allowed us to develop a classification of the entities and roles in the scenario without committing to a specific perspective, and to reconstruct the perspective of, say, a lab technician by looking at its role definition, its associated goals and the activities it performs. The fact that these views are obtained by extracting information within a single formal ontology ensures that these views are coherent (they logically co-exist and do not contradict each other) and aligned in the sense that they relate to the same set of integrated events.
5. *Consistency.* As observed in Section Devising the ontology, consistency is ensured *at development time* by applying ontological analysis to identify the needed categories and by modeling them in the formal language of DOLCE. *At run time* conceptual consistency is preserved because of the clear criteria for classifying the entities in the ontology. Technically, logical consistency is achieved by the computable versions of the ontology, like that in the computational language OWL [6]. There are software environments for managing OWL ontologies, such as Protègè [57]. OWL also supports efficient reasoning so that ontology consistency at run time can be ensured by state-of-the-art automatic inference engines, such as Pellet (freely available for most of the ontology tools [58] and Racer (highly customizable proprietary system) [59].

### Novelties, Impact and Limitations of the study

In this study we showed how to use ontological analysis to develop an ontological model for public institutes in the life sciences. Differently from the literature, we followed a principled top-down approach by starting from a foundational ontology and expanding it via an ontological discussions of the domain. Typically, the opposite is done: one starts from a domain model and aligns it to a foundational ontology. This standard strategy may improve the interoperability of the existing models but does not increase our understanding of the domain nor introduces more flexibility. Instead, we obtained a model rich of new distinctions and that can be used to reason from different perspectives.

Among the advantages of our ontological model, we recall that it has a rich role hierarchy useful to highlight conflicting goals and other dependencies, distinguishes physical objects from their descriptions and their social status, allows multiple views on single processes and makes space for modeling hybrid elements as we showed with knowledge objects.

It is too early to talk about the implementation of this ontological model in the IZSVe information system. The number of required changes is considerable also because ontological models lead to important changes from the data management viewpoint. This is a relevant limitation of our work since actual capacities and advantages can be established only when the system is practically exploited. At the moment, the gained deep understanding of the domain is the most important result we can report about.

### Future work

The work in this paper concentrated mainly on the study of objects and roles in the management of samples and related data from the reception to the release of the analysis’ report. This part of the model needs to be better connected to the analysis of the processes which is still ongoing.

In the future we need to evaluate which parts of the new model are more complex to implement and disruptive with respect to the existing information system. Also, the changes suggested by the new model will likely trigger new requirements and services, which should be evaluated beforehand. Finally, we need to understand how to modularize the model in order to reduce development concerns and to optimize software implementation.

## Conclusions

In this paper we presented elements of a wide-range analysis of processes, data and roles in a large public institute in the life sciences. We showed how to perform an ontological analysis of the domain, what distinctions it highlights, and how to model them in an ontology. This principled approach led us to define a series of notions that together cover a large variety of data, procedures and objects; these are indicative of the complexity of real-life organizations and of the capacity of ontological models approaches to model them.

The result of this work is an ontological framework, technically an extension of DOLCE, which is tuned to the IZSVe scenario.

Finally, we have also exemplified the use of flexible conceptual techniques, via the notion of knowledge object, which help to reconstruct the organization’s perspective within the ontological viewpoint.

## Abbreviations

A: Responsible agent
ASO: Agentive social object
BPMN: Business process model and notation
D: Information entity
DOLCE: Descriptive ontology for linguistic and cognitive engineering
GIS: Geographic information system
GUI: Graphical user interface
IZSVe: Istituto Zooprofilattico Sperimentale delle Venezie
TC: International Organization for Standardization/Technical Committee
LARU: Reception and public relation laboratory
LIMS: Laboratory information management system
LOINC: Logical observation identifiers names and codes
M: Material entity
MDA: Model driven architecture
NPED: Non-physical endurant
KO: Knowledge object
CT: Systematized nomenclature of medicine - clinical terms
UML: Unified modeling language

## References

[1] Gruber TR (1995). Toward principles for the design of ontologies used for knowledge sharing. Int J Hum Comput Stud.

[2] Guarino N, Guarino N (1998). Formal ontology in information systems. Proceedings of the Second International Conference on Formal Ontology in Information Systems.

[3] Bielinis S. How Siri on iPhone 4S works and why it’s a big deal. Apple’s AI tech details in 230 pages of patent app. http://www.unwiredview.com/2011/10/12/howsiri-on-iphone-4s-works-and-why-it%E2%80%99s-a-big-deal-apple%E2. %80\%99s-ai-tech-details-in-230-pagesof-patent-app/. Accessed 1 Sept 2016.

[4] IBM. IBM Watson. http://www.ibm.com/smarterplanet/us/en/ibmwatson/. Accessed 1 Sept 2016.

[5] SAP Community Network. Common Standards - Ontology Definition Languages. http://scn.sap.com/docs/DOC-18520. Accessed 1 Sept 2016.

[6] World Wide Web Consortium. OWL 2 Web Ontology Language Document Overview (Second Edition). http://www.w3.org/TR/owl2-overview/. Accessed 1 Sept 2016.

[7] National Center for Biomedical Ontology: NCBO Bioportal. http://bioportal.bioontology.org/. Accessed 1 Sept 2016.

[8] The Open Biological and Biomedical Ontologies: Open Biomedical Ontologies (OBO) Foundry. http://obofoundry.org. Accessed 1 Sept 2016.

[9] Bodenreider O. Biomedical ontologies in action: role in knowledge management, data integration and decision support. Yearbook Med Inform. 2008:67–79. http://imia.schattauer.de/en/contents/archive/issue/2256/manuscript/9821.html. PMC2592252 18660879

[10] Ashburner M, Ball CA, Blake JA, Botstein D, Butler H, Cherry JM, Davis AP, Dolinski K, Dwight SS, Eppig JT, Harris MA, Hill DP, Issel-Tarver L, Kasarskis A, Lewis S, Matese JC, Richardson JE, Ringwald M, Rubin GM, Sherlock G (2000). Gene ontology: tool for the unification of biology. The Gene Ontology Consortium. Nat Genet.

[11] Rosse C MJ (2003). A reference ontology for biomedical informatics: the foundational model of anatomy. J Biomed Inform.

[12] Hayamizu T, Mangan M, Corradi J, Kadin J, M R (2005). The adult mouse anatomical dictionary: a tool for annotating and integrating data. Genome Biol..

[13] Plant Ontology Consortium: The Plant Ontology Consortium: Plant Ontology (PO). http://www.plantontology.org. Accessed 1 Sept 2016.

[14] Balhoff JP, Dahdul WM, Kothari CR, Lapp H, Lundberg JG, Mabee P, Midford PE, Westerfield M, Vision TJ (2010). Phenex: Ontological annotation of phenotypic diversity. PLoS ONE.

[15] Keet C, Antoniou G, Grobelnik M, Simperl E, Parsia B, Plexousakis D, De Leenheer P, Pan J (2011). The use of foundational ontologies in ontology development: An empirical assessment. The Semantic Web: Research and Applications. Lecture Notes in Computer Science, vol. 6643.

[16] Deus HF, Stanislaus R, Veiga DF, Behrens C, Wistuba II, Minna JD, Garner HR, Swisher SG, Roth JA, Correa AM, Broom B, Coombes K, Chang A, Vogel LH, Almeida JS (2008). A semantic web management model for integrative biomedical informatics. PLoS ONE.

[17] Hoehndorf R, Dumontier M, Oellrich A, Rebholz-Schuhmann D, Schofield PN, Gkoutos GV (2011). Interoperability between biomedical ontologies through relation expansion, upper-level ontologies and automatic reasoning. PLoS ONE.

[18] IZSVe. Istituto Zooprofilattico Sperimentale delle Venezie. http://www.izsvenezie.it/. Accessed 1 Sept 2016.

[19] McDonald CJ, Huff SM, Suico JG, Hill G, Leavelle D, Aller R, Forrey A, Mercer K, DeMoor G, Hook J, Williams W, Case J, Maloney P (2003). Loinc, a universal standard for identifying laboratory observations: a 5-year update. Clin Chem.

[20] Cornet R, de Keizer N. Forty years of snomed: a literature review. BMC medical informatics and decision making. 2008; 8 Suppl 1:S2. 10.1186/1472-6947-8-S1-S2 PMC2582789 19007439

[21] Kenny D (2001). Iso and cen documents on quality in medical laboratories. Clinica Chimica Acta.

[22] Object Management Group. Business Process Model and Notation. http://www.bpmn.org/. Accessed 1 Sept 2016.

[23] Object Management Group. Unified Modeling Language^TM^ (UML^®;^) Resource Page. http://www.uml.org/. Accessed 1 Sept 2016.

[24] Soley R. Model driven architecture. Technical Report 308, 5: EUI Working Papers; 2000.

[25] Gruninger M, Fox M (1995). Methodology for the design and evaluation of ontologies. IJCAI’95, Workshop on Basic Ontological Issues in Knowledge Sharing.

[26] Rector A (1998). Thesauri and formal classifications: terminologies for people and machines. Methods Inf Med.

[27] Mizoguchi R (2004). Tutorial on ontological engineering: Part 2: Ontology development. tools and languages. New Generation Comput.

[28] Guarino N, Welty C, Staab S, Studer R (2004). An overview on ontoclean. Handbook on Ontologies.

[29] Smith B, Ceusters W, Klagges B, Köhler J, Kumar A, Lomax J, Mungall C, Neuhaus F, Rector A, Rosse C (2005). Relations in biomedical ontologies. Genome Biol..

[30] Vieu L, Borgo S, Masolo C, Eschenbach C, Gruninger M (2008). Artefacts and roles: Modeling strategies in a multiplicative ontology. Proceedings of the 5th FOIS Conference.

[31] Smith B. An Introduction to Ontology: From Aristotle to the Universal Core - Training course in eight lectures by Barry Smith. http://ontology.buffalo.edu/smith/IntroOntology_Course.html. Accessed 1 Sept 2016.

[32] Hoehndorf R, Dumontier M, Gkoutos GV (2013). Evaluation of research in biomedical ontologies. Brief Bioinform.

[33] Masolo C, Borgo S, Gangemi A, Guarino N, Oltramari A, Schneider L. The WonderWeb Library of Foundational Ontologies. 2002;17. http://wonderweb.man.ac.uk/deliverables.shtml.

[34] Borgo S, Masolo C, Staab S, Studer R (2009). Foundational Choices in DOLCE. Handbook on Ontologies, 2nd edn. International handbooks on information systems.

[35] Laboratory for Applied Ontology - CNR: DOLCE : a Descriptive Ontology for Linguistic and Cognitive Engineering. http://www.loa.istc.cnr.it/old/DOLCE.html. Accessed 1 Sept 2016.

[36] Masolo C, Vieu L, Bottazzi E, Catenacci C, Ferrario R, Gangemi A, Guarino N, Dubois D, Welty C, Williams MA (2004). Social roles and their descriptions. Proceedings of the 9th International Conference on the Principles of Knowledge Representation and Reasoning (KR).

[37] Borgo S, Vieu L, Meijers A (2009). Artifacts in Formal Ontology. Handbook of the Philosophy of the Technological Sciences. Technology and Engineering Sciences vol. 9.

[38] Ferrario R, Oltramari A, Varzi AC, Vieu L (2004). Towards a computational ontology of mind. Proceedings of the International Conference on Formal Ontology in Information Systems (FOIS 2004).

[39] Semy SK, Pulvermacher MK, Obrst LJ. Toward the use of an upper ontology for u.s. government and u.s. military domains: An evaluation. Technical Report Technical Report MTR 04B0000063. 2004.

[40] Kutz O, Mossakowski T (2011). A modular consistency proof for dolce. Proceedings of the Twenty-Fifth AAAI Conference on Artificial Intelligence.

[41] Boella G, van der Torre L, Verhagen H (2007). Roles, an interdisciplinary perspective. Appl Ontol.

[42] Mizoguchi R, Sunagawa E, Kozaki K, Kitamura Y (2007). The model of roles within an ontology development tool: Hozo. Appl Ontol.

[43] Noy NF, McGuinness DL. Ontology development 101: A guide to creating your first ontology. Technical report 2001. http://protege.stanford.edu/publications/ontology_development/ontology101.pdf. Accessed 2 Sept 2016.

[44] Fernandez-Barrera M, Sartor G. Classifications and the law: Doctrinal classifications vs. computational ontologies. LAW 2010/10. 2010. http://ssrn.com/abstract=1698686. Accessed 1 Sept 2016.

[45] Pozza G, Borgo S, Ravarotto L (2009). From data to knowledge objects, ontological considerations with inputs from the public health domain. 10th European Conference on Knowledge Management.

[46] Borgo S, Pozza G, Ferrario R, Oltramari A (2009). Disentangling knowledge objects. Proceedings of the 4th FOMI Workshop, vol. FAIA 198.

[47] Borgo S, Pozza G (2012). Knowledge objects: a formal construct for material, information and role dependences. Knowl Manag Res Pract.

[48] Loebe F (2007). Abstract vs. social roles–towards a general theoretical account of roles. Appl Ontol.

[49] Arp R, Smith B (2008). Function, role, and disposition in basic formal ontology. Proceedings of Bio-Ontologies Workshop (ISMB 2008).

[50] Mizoguchi R, Kitamura Y, Borgo S. In: International Conference on Formal Ontology in Information Systems (FOIS 2012). IOS Press. p. 103–116.

[51] Keet M. A formal theory of granularity. PhD thesis: Research Centre, KRDB, Faculty of Computer Science, Free University of Bozen-Bolzano; 2008.

[52] Borgo S, Franssen M, Garbacz P, Kitamura Y, Mizoguchi R, Vermaas PE (2014). Technical artifacts: an integrated perspective. Appl Ontol.

[53] Kassel G (2010). A formal ontology of artefacts. Appl Ontol.

[54] Obrst L, Ceusters W, Mani I, Ray S, Smith B. The evaluation of ontologies In: Baker CJO, Cheung KH, editors. Semantic Web. Springer US: 2007. p. 139–158. http://dx.doi.org/10.1007/978-0-387-48438-9_8.

[55] Rector AL, Qamar R, Marley T (2009). Binding ontologies and coding systems to electronic health records and messages. Appl Ontol.

[56] Jansen L SS. The ten commandments of ontological engineering In: Herre H, Hoehndorf R, Loebe F, editors. OBML 2011 Workshop Proceedings. Leipzig, Germany: Markus Loeffler. p. 11.

[57] Knublauch H, Fergerson R, Noy N, Musen M. The protégé owl plugin: An open development environment for semantic web applications In: McIlraith S, Plexousakis D, van Harmelen F, editors. The Semantic Web–ISWC 2004. Springer: 2004. p. 229–43.

[58] GitHub: Pellet: An Open Source OWL DL reasoner for Java. https://github.com/complexible/pellet. Accessed 2 Sept 2016.

[59] Institute of Information Systems: RACER. http://www.ifis.uni-luebeck.de/~moeller/racer/. Accessed 2 Sept 2016.

